# Mechanical Behaviour of Multifunctional Epoxy/Hollow Glass Microspheres/Paraffin Microcapsules Syntactic Foams for Thermal Management

**DOI:** 10.3390/polym13172896

**Published:** 2021-08-27

**Authors:** Francesco Galvagnini, Giulia Fredi, Andrea Dorigato, Luca Fambri, Alessandro Pegoretti

**Affiliations:** INSTM Research Unit, Department of Industrial Engineering, University of Trento, Via Sommarive 9, 38123 Trento, Italy; giulia.fredi@unitn.it (G.F.); andrea.dorigato@unitn.it (A.D.); luca.fambri@unitn.it (L.F.)

**Keywords:** syntactic foams, epoxy, glass microspheres, thermal energy storage, phase change materials, thermal properties

## Abstract

Epoxy/hollow glass microsphere (HGM) syntactic foams (SFs) are peculiar materials developed to combine low density, low thermal conductivity, and elevated mechanical properties. In this work, multifunctional SFs endowed with both structural and thermal management properties were produced for the first time, by combining an epoxy matrix with HGM and a microencapsulated phase change material (PCM) having a melting temperature of 43 °C. Systems with a total filler content (HGM + PCM) up to 40 vol% were prepared and characterized from the mechanical point of view with a broad experimental campaign comprising quasi-static, impact, and fracture toughness tests. The experimental results were statistically treated and fitted with a linear model, to produce ternary phase diagrams to provide a comprehensive interpretation of the mechanical behaviour of the prepared foams. In quasi-static tests, HGM introduction helps to retain the specific tensile elastic modulus and to increase the specific compressive modulus. The brittle nature of HGMs decreases the Charpy impact properties of the SFs, while the PCM insertion improve their toughness. This result is confirmed in K_IC_ and G_IC_ tests, where the composition with 20 vol% of PCM shows an increase of 80% and 370% in K_IC_ and G_IC_ in to neat epoxy, respectively. The most promising compositions are those combining PCM and HGMs with a total particle volume fraction up to 40 vol%, thanks to their optimal combination of thermal management capability, lightness, thermal insulation, and mechanical properties. The ability to fine-tune the properties of the SFs, together with the acquired thermal energy storage (TES) capability, confirm the great potential of these multifunctional materials in automotive, electronics, and aerospace industries.

## 1. Introduction

Phase change materials (PCMs) have been increasingly utilized for thermal energy storage (TES) and thermal management (TM). Given their ability to store and release heat at a nearly constant temperature, PCMs can reduce the gap between thermal energy need and availability [1,2,3]. Hence, they can be used to manage natural energy resources more efficiently and can be exploited to temporarily store excess heat in solar-thermal power plants or solar-thermal systems for indoor temperature regulation and water heating [4,5,6]. PCMs have also been used for thermal management in buildings, smart textiles, electronic components, and electric vehicle batteries [7,8,9,10], as they can maintain the temperature in a controlled range during phase change. The most diffused PCMs working at low-medium temperature (0–120 °C) are organic solid–liquid oligomers such as paraffins, poly(ethylene glycol)s (PEGs), and fatty acids and alcohols. They have a large latent heat of fusion (up to 250 J/g) and a tunable working temperature, and they are lightweight, inexpensive, and chemically inert [11,12,13]. On the other hand, their main shortcoming is their need for confinement above the melting temperature, to avoid leakage and loss of material [2,14]. This problem is generally addressed by encapsulating PCMs into macro-, micro-, or even nano-containers, which also (i) prevent undesired interactions between the PCM and the surrounding environment, (ii) accommodate the volume variation during phase change, and (iii) improve the heat transfer and the thermal stability [15,16,17]. 

PCMs are also interesting as they allow the design of multifunctional composite materials performing both structural and heat management functions. In fact, in most applications requiring thermal management, the TES property is generally attributed to a monofunctional supplementary module, e.g., computer fans or finned structures. This approach naturally increases the weight and volume of the whole component, and this is generally an undesired side effect, especially when light-weight design is recommended as for applications in the automotive and portable electronics fields. A possible strategy to overcome this issue is to embed the TES/TM function within the structural elements, and this can be achieved by building the structure with multifunctional materials that can simultaneously carry load and manage heat. The best candidates for this aim, among all classes of materials, are polymer composites, as they combine the properties of a tough and lightweight matrix with those of structural and functional fillers [18]. In this perspective, PCMs can be used as a functional filler in combination with a reinforcing agent, to obtain a multifunctional composite with balanced structural and TES properties. Our group has recently investigated this concept by developing several polymer/PCM/reinforcement systems, employing mostly paraffinic PCMs combined with thermoplastic or thermosetting matrices and continuous or discontinuous reinforcing fibers [19,20,21,22,23,24,25,26,27,28,29]. 

More recently, our group has introduced paraffin microcapsules in another type of polymer composite, i.e., an epoxy/hollow-glass-microspheres syntactic foam [30]. Syntactic foams (SFs) are closed-cell porous materials constituted by a continuous phase (generally polymeric) and rigid hollow particles, and this particular microstructure allows de-creasing density and improved mechanical properties compared to bulk or traditionally foamed polymers. The elevated mechanical performance per unit mass is coupled with other interesting functional properties, such as thermal, electrical, and acoustic insulation and fire resistance [31]. For this reason, they could be interesting for high-end, weight-critical applications in the aerospace, transportation, and marine fields [32,33,34,35]. 

Both components of SFs, i.e., the matrix and hollow particles, can be made of a wide variety of polymeric, ceramic, or metallic materials [34,36]. The most widely investigated SFs are those composed of epoxy matrix and hollow glass microspheres (HGMs) [32,37,38,39,40,41], due to their versatile combination of high thermo-mechanical properties and low density. In fact, their properties can be tailored by varying the HGM diameter, size distribution, volume fraction, shell thickness, surface morphology, and surface reactivity. Generally, high HGM concentrations and thin shells result in materials with low density but limited stiffness and mechanical and impact strength, while the specific (i.e., normalized by density) mechanical properties often increase with the HGM volume fraction [42,43,44]. The property set of these SFs can be further expanded by incorporating a third phase, such as short carbon or glass fibers, carbon nanotubes, and nanoclays, which can enhance the fracture toughness by modifying the packing density of HGMs, and can add other functional properties such as thermal conductivity and electromagnetic interference (EMI) shielding capability [33,45,46].

Our group has recently produced epoxy/HGM SFs containing, as a third phase, a microencapsulated paraffinic PCM with a melting temperature of 43 °C [30]. In that paper, a comprehensive microstructural and thermal characterization of these foams was carried out. However, despite the promising properties of these systems, a complete and detailed mechanical characterization, fundamental to fully understand the application range of these systems, is still missing. Hence, this work aims at elucidating the mechanical behavior of these peculiar multifunctional syntactic foams. In particular, this work focuses on fifteen formulations with different HGM-to-PCM ratios and a total filler content (HGM + PCM) up to 40 vol%. Composites were prepared and characterized with a wide range of mechanical characterization techniques including tensile, compressive, Charpy, and mode I fracture toughness tests.

## 2. Materials and Methods

### 2.1. Materials

The epoxy resin, kindly provided by Elantas Europe Srl (Collecchio, PR, Italy), was a bi-component mixture composed of an epoxy base EC157 (density = 1.15 g/cm^3^, viscosity at 25 °C = 600–800 mPa⋅s) and a hardener W342 (density = 0.95 g/cm^3^, viscosity at 25 °C = 30–70 mPa⋅s). K15 hollow soda-lime-borosilicate glass microspheres (HGMs) were provided by 3M Italia Srl (Pioltello, Italy). They had a density of 0.15 g/cm^3^, a mean particle size (D_50_) of 60 μm, a crush strength (90% survival) of 2.07 MPa, and a thermal conductivity of 0.055 W/(m∙K). A microencapsulated paraffin MPCM43D (Microtek laboratories Inc., Dayton, OH, USA), composed of a paraffinic core and a melamine-formaldehyde shell, constituting about the 15 wt% of the PCM, was utilized. This PCM had a melting enthalpy of 190–200 J/g, a melting temperature of 43 °C, mean size of 17–22 µm, and a density of 0.9 g/cm^3^. Both HGMs and the PCM were used as received.

### 2.2. Sample Preparation

**Figure 1:**
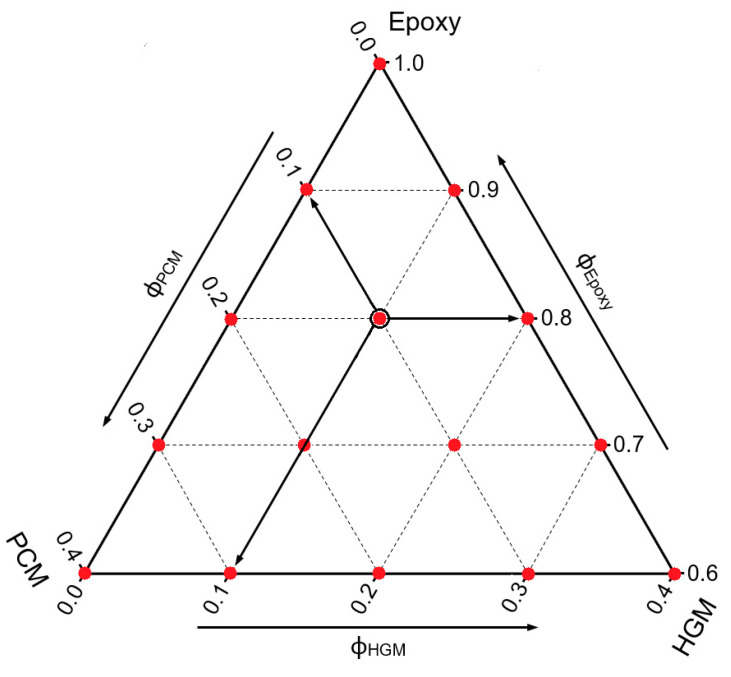
Graphical representation of the prepared compositions (red dots) on the ternary diagram. As example of graph legend, the black-bordered dot refers to the EPG-10.10 foam, having a PCM concentration of 10 vol% and a HGM concentration of 10 vol%.

Epoxy/HGM/PCM syntactic foams were prepared by mixing the epoxy base, the PCM, and HGMs in a becker for 5 min at 100 rpm by using a Dispermat F1 mechanical mixer (VMA-Getzmann GmbH, Reichshof, Germany), and the resulting mixtures were then degassed through a vacuum pump for 5 min. After this step, the hardener was added and the mixing and degassing operations were repeated. The mixtures were then cast in silicone molds, cured at room temperature for 24 h, and post-cured in an oven at 80 °C for 6 h. Samples were labeled as EPG-x.y, where x represents the PCM volume content and y the HGM concentration, both ranging between 0 vol% and 40 vol%. The fifteen compositions selected in this work, having a maximum total filler content of 40 vol%, are listed in Table 1 and reported on the ternary diagram shown in Figure 1, where the prepared formulations are indicated with red dots. Moreover, Figure 1 gives a general example of how a ternary phase diagram should be read.

### 2.3. Experimental Techniques

#### 2.3.1. Mechanical Characterization

All mechanical tests were performed at 25 °C and 50% of relative humidity. Quasi-static tensile tests were carried out by using an Instron 4502 testing machine (Instron, Turin, Italy) equipped with a 10 kN load cell, following the ISO-524-2 standard. Ten type-1B specimens were tested for each composition to determine firstly the tensile elastic modulus (E_t_) and secondly the stress at break (σ_B_). The tests for measuring the elastic modulus were all conducted with an extensometer with a gauge length of 50 mm, at a crosshead speed of 0.5 mm/mm, until 0.8% of strain. The steepest tangent line to the curve was used to determine the elastic modulus. The tests for determining the strain at break were all conducted by measuring the strain with the crosshead displacement and a gauge length of 115 mm (distance between grips) at a crosshead speed of 2 mm/min until the failure of the specimens.

Compression tests were carried out according to the ASTM-D695 standard by using an Instron 5969 testing machine equipped with a 50 kN load cell. The elastic modulus at compression (E_C_) and the stress at 10% of strain (σ_10_) were evaluated by testing cylindrical specimens (diameter 20 mm, height 40 mm) at a crosshead speed of 1.3 mm/min. The elastic modulus was determined from the slope of the line tangent to the steepest linear part of the stress–strain curve. Ten specimens were tested for each composition. 

Charpy impact tests were carried out following the ISO 179-2 standard with a Ceast 3549/000 pendulum impact testing machine (Instron, Turin, Italy). The hammer used in this work was set to a starting angle of 51°, resulting in a potential energy of 1 J and an impact velocity of 1.29 m/s. The test was performed on single-notch rectangular specimens with dimensions 80 × 10 × 4 mm^3^, a notch depth of 2 mm, and a notch tip radius of 0.25 mm. At least 10 specimens, having a span length of 62 mm, were tested for each composition. In this way, it was possible to determine the specific energy absorbed under impact conditions (a_cN_). 

The plane-strain fracture toughness (K_IC_) and the critical strain energy release rate (G_IC_) of the prepared foams were determined following the ASTM D5045 standard, with an Instron 5969 testing machine equipped with a 1 kN load cell. For K_IC_, single-edge notched specimens with dimensions of 50 × 12 × 6 mm^3^ were tested in a three-point bending configuration, at a crosshead speed of 10 mm/min. The support span was four times the width (48 mm). The notch was produced by sawing the specimens with a razor blade until reaching their half-width. According to ASTM D5045 standard, G_IC_ was determined taking the system compliance into account, as determined from tests on unnotched specimens. At least 10 specimens were tested for each composition.

#### 2.3.2. Design of Experiment (DOE) and Statistical Analysis of the Experimental Data

Due to the wide variety of possible compositions in a ternary system, the analysis of the properties can be a very complex process. As already demonstrated in our previous work on these SFs [30], preliminary and post-production statistical approaches can be very useful to set up experiments and analyze the results. In this paper, a statistical approach based on a mixture design was implemented to define the most representative compositions and to represent the results with ternary phase diagrams. For this purpose, the “mixexp” package was used in the RStudio v.1.4.1103 software (RStudio, Inc., Boston, MA, USA) to perform the mixture design, while the “lm” function was used to fit by a quadratic linear model called “Scheffé quadratic model” [47] (see Equation (1)) the experimental results:

(1) $$y=\overset{q}{\underset{i=1}{\sum }}\beta_{i}x_{i}+\overset{q-1}{\underset{i=1}{\sum }}\overset{q}{\underset{j=i+1}{\sum }}\beta_{\mathrm{ij}}x_{i}x_{j}+\epsilon$$

where y is the response variable, x_i_ and x_j_ are the binary mixture compositions, β_i_ represents the expected response at the vertex, and β_ij_ are the coefficients indicating the amount of the quadratic curvature along the edge of the simplex region [47]. After a first fit, the most significant components of the model (x_i_, x_j_) were evaluated through the analysis of variance (ANOVA). At this point, all non-significant components and combinations were removed from the model and a new fit with the corrected model was performed. This procedure was repeated until only the statistically significant terms remained. At this point, the model could be considered statistically correct and therefore used to represent the analyzed data. The function “ModelPlot” was used to represent the ternary models, and each plot also reported the resulting R^2^_adj_ and the average coefficient of variance ACV of the fitting model (see Equation (2)):

(2) $$\mathrm{ACV}=\frac{1}{N}\overset{N}{\underset{j=1}{\sum }}{\mathrm{CI}}_{j}^{\mathrm{Rel}}$$

where N is the number of compositions considered for that test (15 in this case), and ${\mathrm{CI}}_{j}^{\mathrm{Rel}}\;$ is the relative confidence interval of the j-th composition (sample), defined as reported in Equation (3):

(3) $${\mathrm{CI}}_{j}^{\mathrm{Rel}}=\left(\frac{{\mathrm{CI}}_{j}}{\bar{x_{j}}}\right)\cdot 100$$

where $\bar{x_{j}}$ is the average value of the test values of the j-th sample and CI_j_ is the confidence interval of a test of the j-th composition (sample), defined via Equation (4):

(4) $${\mathrm{CI}}_{j}=t_{j}\cdot \frac{s_{j}}{\sqrt{n_{j}}}$$

where t_j_ is the t-value calculated from the t distribution for the j-th sample of a test, n_j_ is the number of specimens of the j-th sample, and s_j_ is the standard deviation of the results of the j-th sample, defined in Equation (5):

(5) $$s_{j}=\sqrt{\frac{\sum_{i=1}^{n_{j}}{\left(x_{\mathrm{ji}}-\bar{x_{j}}\right)}^{2}}{n_{j}-1}}$$

where x_ji_ is the measured value of that test of the i-th specimen of the j-th sample, and $\bar{x_{j}}$ is the average value of the test of the j-th sample.

## 3. Results and Discussion

### 3.1. Quasi-Static Tensile Properties

**Figure 2:**
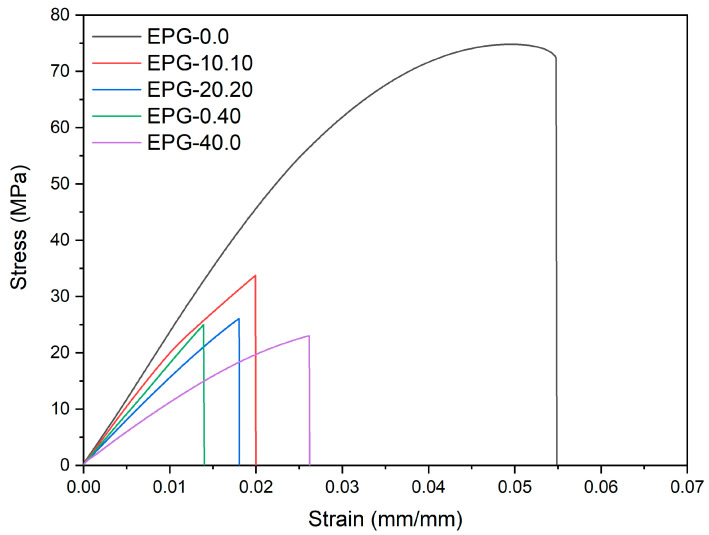
Representative stress–strain curves from quasi-static tensile tests of five selected syntactic foams.

**Figure 3:**
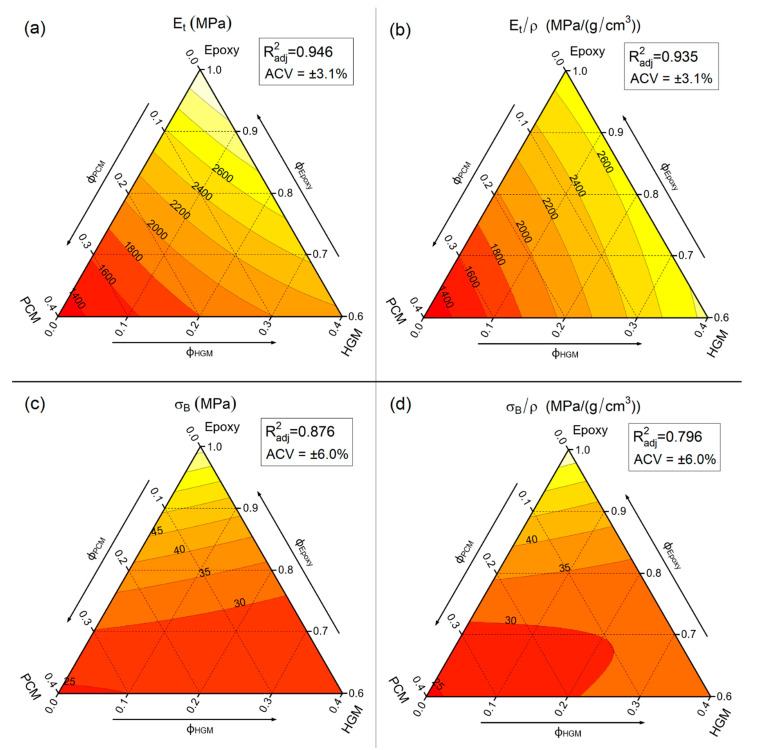
Fit-models of the main results of quasi-static tensile tests of the prepared syntactic foams. (**a**) Young’s modulus (E_t_); (**b**) specific Young’s modulus (E_t_/ρ); (**c**) tensile stress at break (σ_B_); and (**d**) specific stress at break (σ_B_/ρ). R^2^_adj_ = adjusted R-squared, ACV = average coefficient of variance.

One of the most widely used methods to evaluate the mechanical properties of polymer-based systems is the uniaxial quasi-static tensile test. Figure 2 represents the stress–strain curves of some selected compositions, while Figure 3 summarizes the trends of tensile elastic modulus (E_t_), specific elastic modulus (E_t_/ρ), stress at break (σ_B_), and specific stress at break (σ_B_ /ρ) through ternary diagrams representing the linear fit model of the obtained experimental results (see Section 2.3).

Figure 2 and Figure 3 evidence the role played by both filler types (i.e., PCM and HGM) on the tensile properties. Compared to neat epoxy resin, the elastic modulus, strength, and strain at break of all composites are considerably lower. The elastic modulus, which is reduced especially by the PCM, decreases from 3193 ± 99 MPa down to 1288 ± 40 MPa for the EPG-40.0 sample (−60%). For the same formulation, the stress at break is reduced from 73.7 ± 1.8 MPa down to 22.4 ± 0.8 MPa (−70%), while the strain at break decreases from 5.7 ± 0.5% down to 2.5 ± 0.3% (−56%). These results are in good agreement with our previous findings on epoxy/PCM composites [48]. These trends are also evidenced by the application of the linear model (see Figure 3a–d). The elastic modulus (Figure 3a) decreases especially upon PCM addition, while a less evident effect can be observed with HGMs, as the stiffness for the sample containing 40 vol% of HGMs is 2124 ± 55 MPa (−32% than neat epoxy). Considering the compositions with both HGMs and PCM at constant total vol%, moving horizontally on the ternary graph, it can be concluded that the gradual substitution of PCM with HGMs increases the elastic modulus, due to the higher stiffness of HGMs compared to the PCM capsules. For example, considering the compositions with a total filler concentration of 30 vol%, EPG-30.0 foam shows an elastic modulus of 1626 ± 50 MPa, the EPG-20.10 foam of 1936 ± 60 MPa, the sample EPG-10.20 of 2207 ± 68 MPa, and EPG-0.30 of 2438 ± 76 MPa.

This effect is even more evident considering the specific modulus (E_t_/ρ), reported in Figure 3b. By moving horizontally on the graph from left to right, the increase in specific modulus at constant filer volume fraction is still evident. Moreover, the specific modulus shows a strong dependency only on the PCM amount. In fact, by maintaining a constant PCM concentration, the specific modulus remains nearly constant as the HGM content increases. This results from the fact that the HGMs decrease the elastic modulus but also considerably decrease the density, thereby maintaining the E_t_/ρ ratio nearly constant. This result is important from a design point of view, as it clearly shows that the HGMs decrease the system density without impairing its specific stiffness. 

Concerning the mechanical strength, Figure 3c shows that the introduction of either PCM or HGMs decreases the stress at break. In compositions containing only the PCM, the strength decreases with an increase in the PCM fraction, and for the sample EPG-40.0 is close to −65% compared to neat epoxy resin. A similar trend can be noticed for samples containing only HGMs. The normalization by density does not substantially modify these trends, as reported in Figure 3d. As already explained in our previous work on these systems [30], this reduction in the mechanical strength can be attributed to the creation of porosities due to HGMs insertion, and also to the limited HGM/epoxy interfacial adhesion [49,50,51,52]. A possible way to overcome this issue is the surface functionalization of the HGMs by silanization [53], which will be the object of an upcoming work.

### 3.2. Compressive Properties

**Figure 4:**
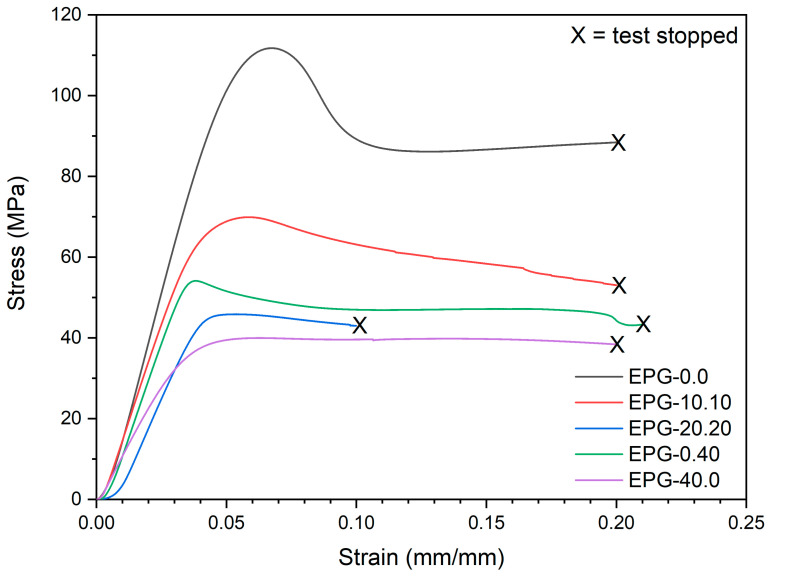
Representative stress–strain curves from quasi-static compressive tests on the prepared syntactic foams.

**Figure 5:**
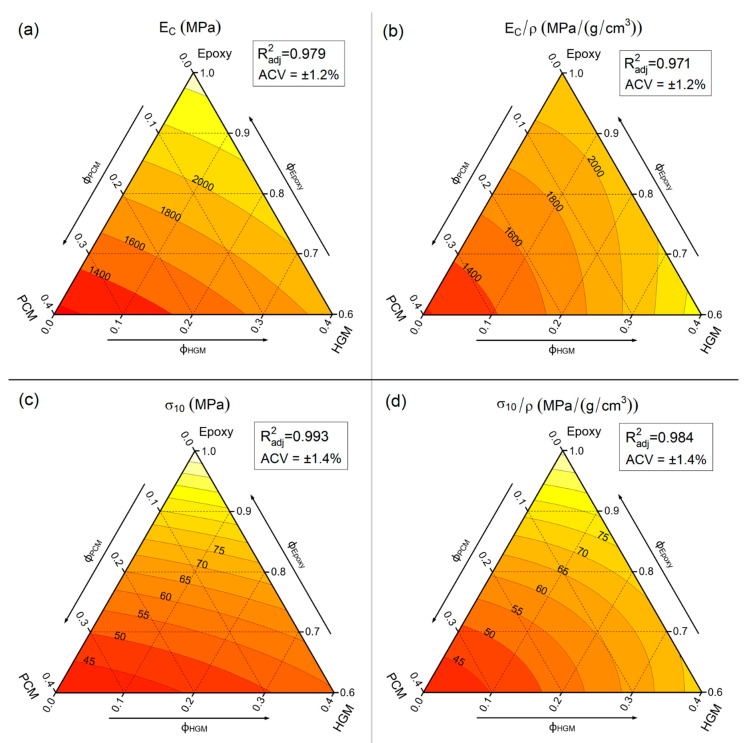
Fit-models of the main results of quasi-static compressive tests on the prepared syntactic foams. (**a**) compressive modulus (E_c_); (**b**) specific compressive modulus (E_c_/ρ); (**c**) compressive stress at a strain of 10% (σ_10_); and (**d**) specific compressive stress at a strain of 10% (σ_10_/ρ). ACV average coefficient of variance. R^2^_adj_ = adjusted R-squared, ACV = average coefficient of variance.

Another widely used mechanical test to analyze the properties of syntactic foams is the compression test, as tremendous importance has always been given in practical applications to the compressive resistance of syntactic foams [54,55,56]. Figure 4 reports the compressive stress–strain curves of some selected compositions, while Figure 5a–d show the trends of the compression modulus (E_C_), specific compression modulus (E_C_/ρ), stress at 10% of strain (σ_10_), and specific stress at 10% of strain (σ_10_/ρ).

The representative stress–strain curves (Figure 4) evidence that the compressive properties decrease upon the addition of both PCM and HGMs. The compressive modulus (E_C_) decreases from 2461 ± 29 MPa of neat epoxy down to 1172 ± 16 MPa of the sample EPG-40.0 (−53%), while σ_10_ is reduced from 109.9 ± 1.3 MPa of neat epoxy down to 40.0 ± 0.6 MPa of the sample EPG-40.0 (−63%). If the compressive curves with the same filler amount are considered, e.g., EPG-20.20, EPG-0.40, and EPG-40.0, a similar trend can be detected, especially after the yield point. 

The quantitative trends of the investigated mechanical properties can be observed in Figure 5a–d. Similar to the tensile modulus, E_C_ also decreases more markedly due to PCM insertion rather than to HGMs addition. In fact, in compositions containing only the PCM, E_C_ decreases from 2484 ± 30 MPa of neat epoxy to 1149 ± 14 MPa of EPG-40.0 (−54%), while in compositions containing only HGM the E_C_ decreases by only 24%, from 2484 ± 30 MPa of neat epoxy to 1885 ± 23 MPa of EPG-0.40. Moreover, the progressive substitution of PCM with HGMs increases the stiffness of the system. Conversely, the shape of both E_T_ and E_C_ level lines slightly differ from that observed in tensile tests. For compositions containing only HGMs, the elastic modulus decreases with the HGM amount, but E_C_ is less affected than E_t_. This difference is even more evident by looking at the trends of specific compressive modulus (Figure 5b), where the maximum value is not shown by the neat epoxy, as in the tensile test, but by the EPG-0.40 sample. This is one of the most important reasons why syntactic foams are mainly used in applications where a compression state is applied, as their combination of low density and good compressive stiffness results in a very high specific compressive modulus. 

For the compressive strength, the stress at 10% of strain (Figure 5c) decreases with the total filler amount. By looking at the PCM-only containing samples, the reduction in σ_10_ is approx. 65%, from 110 MPa of the neat epoxy resin down to 40 MPa of the EPG-40.0 foam. This reduction is slightly attenuated for HGM-only filled samples (approx. 50% for EPG-0.40). On the other hand, the normalization by density bends the level curves (Figure 5d) and σ_10_/ρ values are interesting also for compositions with a mid-high amount of fillers.

In conclusion, the better compressive properties of HGMs compared to the PCM [20,50] and the less concerning effect of the poor epoxy/HGMs adhesion in compression [57] result in higher E_C_ and σ_10_ performance of HGM-only filled samples compared to those containing also PCM. This effect is even more evident if the normalized properties are considered. 

### 3.3. Charpy Impact Properties

**Figure 6:**
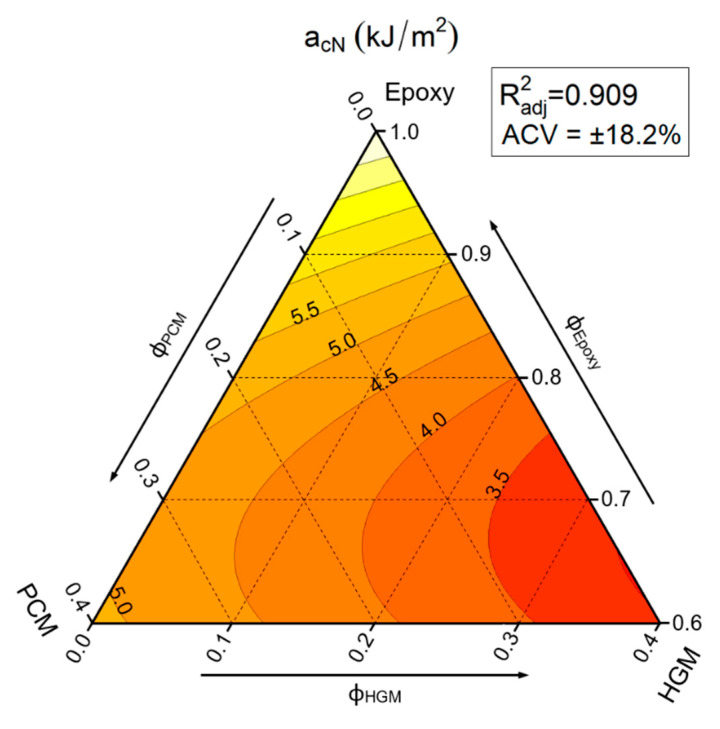
Fit-models of the Charpy impact strength a_cN_ values of the prepared syntactic foams. R^2^_adj_ = adjusted R-squared, ACV = average coefficient of variance.

Figure 6 reports the ternary diagrams representing the linear model fitting of the values of the Charpy impact strength (can) of the prepared foams.

The large ACV (± 18.2%) obtained through the application of the linear fit model indicates a large scatter of the measured a_CN_ values, which is quite common in Charpy impact tests [44]. As it could be expected, Figure 6 shows that HGM filled composites denote a reduction in impact strength due to the fragile nature of HGM and the presence of voids within the material, while PCM, due to its plastic nature, can limit the decrease in impact properties. In particular, by looking at the equi-filled compositions (i.e., moving horizontally on the graph) it is evident how the substitution of HGMs with PCM increases noticeably the impact strength (from 3.12 ± 0.57 kJ/m^2^ of EPG-0.30 sample up to 4.87 ± 0.89 kJ/m^2^ of the EPG-30.0 foam). The samples containing only PCM show a decreasing trend comparable with that reported in our previous work on epoxy/PCM composites [48]. On the other hand, HGMs decrease the impact strength more at lower HGM content (until 30 vol%) compared to higher HGM content. In fact, from 8.06 ± 1.47 KJ/m^2^ of neat epoxy, the impact strength decreases more in a first step down to 3.98 ± 0.72 KJ/m^2^ of EPG-0.20, and less subsequently to 3.04 ± 0.55 KJ/m^2^ of EPG-0.40. These results highlight that the incorporation of both fillers generates a general decrease in impact strength, but the extent of the observed a_CN_ drop depends on the filler type. In any case, it can be supposed that the impact properties of these foams could also be considerably improved by increasing the adhesion between HGMs and the epoxy matrix.

### 3.4. Fracture Behaviour

**Figure 7:**
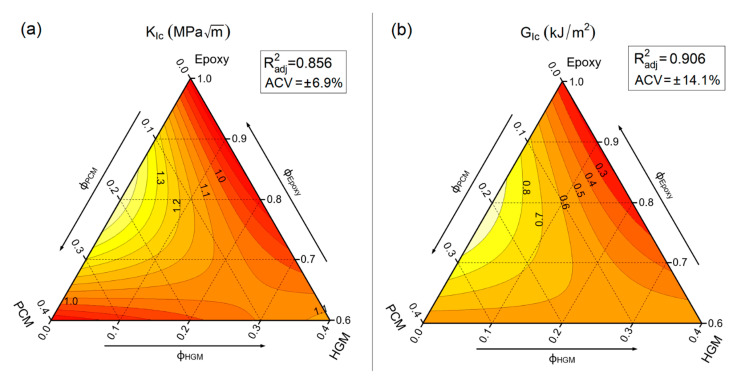
Fit-models of the main results from fracture toughness tests on the prepared syntactic foams. (**a**) mode I fracture toughness (K_IC_) and (**b**) critical strain energy release rate (G_IC_). R^2^_adj_ = adjusted R-squared, ACV = average coefficient of variance.

It is well known that both critical stress intensity factor (K_IC_) and critical strain energy release rate (G_IC_) describe the capability of the material to resist crack initiation. Both properties were investigated, and the results are represented by ternary graphs in Figure 7a,b.

Both K_IC_ and G_IC_ are maximized for the sample containing 20 vol% of PCM (EPG-20.0). The results obtained for PCM-only filled compositions agree with those found in a previous work of our group [48], where the incorporation of PCM microcapsules increased both K_IC_ and G_IC_ up to a PCM content of 20 wt%, and for higher microcapsule contents both properties decreased. On the other hand, HGM gives little contribution to K_IC_ and G_IC_, with a slight increase only at elevated HGM amounts. K_IC_ and G_IC_ for neat epoxy result 0.83 ± 0.06 MPa∙m^1/2^ and 0.20 ± 0.03 kJ/m^2^, respectively, while EPG-20.0 shows values of K_IC_ and G_IC_ increased of about 80% and 370%, respectively, reaching 1.44 ± 0.10 MPa∙m^1/2^ and 0.95 ± 0.13 kJ/m^2^. On the other hand, EPG-40.0 shows values close to that of the unfilled epoxy resin. As shown by SEM micrographs reported in our previous work on these systems [30], this behavior could be explained by the introduction of new toughening mechanisms due to PCM insertion, such as crack pinning, debonding, and microcracking.

### 3.5. General Comparison of the Prepared Syntactic Foams

**Figure 8:**
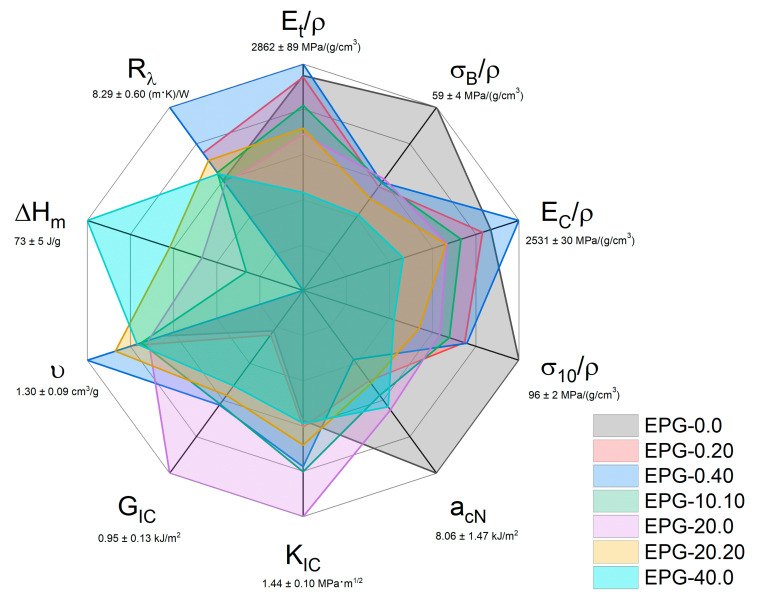
Graphical representation of the properties of some representative compositions analyzed in this paper and in our previous work on these foams [30].

To compare and rank the different compositions of these new ternary systems in terms of thermal and mechanical properties, a radar graph was reported in Figure 8. It compares seven representative compositions over ten selected properties, i.e., specific tensile modulus (E_t_/ρ), specific tensile stress at break (σ_B_/ρ), specific compression modulus (E_C_/ρ), specific compression stress at 10% of strain (σ_10_/ρ), impact strength (a_cN_), mode I fracture toughness (K_IC_), critical strain energy release rate (G_IC_), specific volume (υ = 1/ρ), specific melting enthalpy (ΔH_m_, see ref. [30]), and thermal resistivity (R_λ_ = 1/λ, see ref. [30]). The maximum measured values of each property are reported below each axis lable.

As expected, neat epoxy resin (EPG-0.0) shows the highest values in tensile, compressive, and impact properties, but the poorest performance on the other properties. It combines the highest value of impact strength and the lowest values of K_IC_ and G_IC_. This sample also shows the lowest specific volume, enthalpy of fusion, and thermal resistance. The HGM-only filled samples (EPG-0.20, red and EPG-0.40, blue) cover very different areas of this plot compared to the unfilled resin. They cover smaller areas than EPG-0.0 in the tensile, compression, and impact properties, denoting a general reduction in performance, with some notable exceptions such as the specific compression modulus for the EPG-0.40 foam. On the other hand, these compositions show higher K_IC_, G_IC_, υ, and R_λ_, than neat epoxy. The area covered by PCM-only filled samples (i.e., EPG-20.0 and EPG-40.0 foams) is even smaller than that of the HGM-only filled samples in the tensile, compression, and impact properties, for which the worst composition is EPG-40.0. Conversely, EPG-20.0 is the best composition in terms of K_IC_ and G_IC_, and EPG-40.0 also performs better than EPG-0.0. Of course, the PCM gives TES capabilities to these compositions, which show high values of ΔH_m_. The property set of these compositions privileges the TES properties and underperforms in the mechanical properties, and therefore they are recommended for cavity filling with TES capabilities. The combination of the PCM and HGMs in the EPG-10.10 and EPG-20.20 samples allows reaching a good balance between mechanical and TES properties. The area of these samples is more circular than the others, denoting a more equilibrate property distribution. While EPG-10.10 shows slightly better mechanical properties, EPG-20.20 features better TES performance. These selected compositions represent a good compromise between HGM-only and PCM-only formulations, and thus they can be considered the most promising ones in terms of multifunctionality.

## 4. Conclusions

In this work, the incorporation of HGMs and PCM microcapsules into an epoxy resin resulted in syntactic foams with an interesting combination of properties. HGMs brought a considerable decrease in density, which resulted in increased specific mechanical properties. In fact, the HGMs helped to retain the specific tensile elastic modulus, while the specific compressive modulus was even higher than that of neat epoxy resin for HGM contents higher than 20 vol%. On the other hand, the PCM decreased all specific tensile and compressive properties. An opposite trend was evidenced in Charpy impact tests, where the brittle HGMs considerably decreased the impact properties, whereas the PCM microcapsules were capable to restrain this reduction, probably thanks to their better deformability. Interesting trends were also observed for K_IC_ and G_IC_. In samples containing only the PCM, both K_IC_ and G_IC_ showed a maximum at a PCM content of 20 vol%, denoting a high toughening effect of PCM. HGMs also increased both K_IC_ and G_IC_, but only at elevated concentrations above 20 vol%. 

This work showed that the combination of PCM and HGMs resulted in multifunctional materials with a promising combination of TES capability and specific mechanical properties. The resulting property set can be finely tuned simply by changing the filler relative fractions, which evidences noticeable adaptability to different applications in the electronic, automotive, refrigeration, and aerospace industries. The mechanical performance of these foams could be significantly increased by improving the interfacial adhesion between HGMs and the matrix, thereby allowing a further expansion of the applicability of these materials, which will be the object of future research.

## Figures and Tables

**Table 1:** List and code of the prepared syntactic foams with their relative composition of epoxy matrix, phase change material (PCM), and hollow glass microsphere (HGM).

Sample	Epoxy (vol/%)/(wt/%)	PCM (vol/%)/(wt/%)	HGM (vol/%)/(wt/%)
EPG-0.0	100.0/100.0	0.0/0.0	0.0/0.0
EPG-0.10	90.0/98.5	0.0/0.0	10.0/1.5
EPG-0.20	80.0/96.7	0.0/0.0	20.0/3.3
EPG-0.30	70.0/94.5	0.0/0.0	30.0/5.5
EPG-0.40	60.0/91.6	0.0/0.0	40.0/8.4
EPG-10.0	90.0/91.6	10.0/8.4	0.0/0.0
EPG-10.10	80.0/89.3	10.0/9.2	10.0/1.5
EPG-10.20	70.0/86.5	10.0/10.1	20.0/3.4
EPG-10.30	60.0/83.0	10.0/11.3	30.0/5.7
EPG-20.0	80.0/83.0	20.0/17.0	0.0/0.0
EPG-20.10	70.0/79.7	20.0/18.7	10.0/1.6
EPG-20.20	60.0/75.8	20.0/20.7	20.0/3.5
EPG-30.0	70.0/74.0	30.0/26.0	0.0/0.0
EPG-30.10	60.0/69.8	30.0/28.6	10.0/1.6
EPG-40.0	60.0/64.6	40.0/35.4	0.0/0.0

## Data Availability

The data that supports the findings on this study are available on request by the corresponding author.
